# Movement characteristics during customized exergames after total knee replacement in older adults

**DOI:** 10.3389/fspor.2022.915210

**Published:** 2022-07-27

**Authors:** Maarit Janhunen, Antti Löppönen, Simon Walker, Taavi Punsár, Niina Katajapuu, Sulin Cheng, Juha Paloneva, Konsta Pamilo, Mika Luimula, Raija Korpelainen, Timo Jämsä, Ari Heinonen, Eeva Aartolahti

**Affiliations:** ^1^Faculty of Sport and Health Sciences, University of Jyväskylä, Jyväskylä, Finland; ^2^Faculty of Sport and Health Sciences, Gerontology Research Center, University of Jyväskylä, Jyväskylä, Finland; ^3^Department of Movement Sciences, Physical Activity, Sports and Health Research Group, Katholieke Universiteit Leuven, Leuven, Belgium; ^4^Faculty of Sport and Health Sciences, NeuroMuscular Research Center, University of Jyväskylä, Jyväskylä, Finland; ^5^Health and Well-being, Turku University of Applied Sciences, Turku, Finland; ^6^Exercise Translational Medicine Center, Shanghai Center for Systems Biomedicine, Shanghai Jiao Tong University, Shanghai, China; ^7^Department of Orthopedics and Traumatology, Hospital Nova of Central Finland, Jyväskylä, Finland; ^8^Faculty of Health Sciences, University of Eastern Finland, Kuopio, Finland; ^9^Department of Orthopedics, Coxa Hospital for Joint Replacement, Tampere, Finland; ^10^Faculty of Business and Engineering, Turku University of Applied Sciences, Turku, Finland; ^11^Department of Sports and Exercise Medicine, Oulu Deaconess Institute Foundation sr., Oulu, Finland; ^12^Faculty of Medicine, Center for Life Course Health Research, University of Oulu, Oulu, Finland; ^13^Medical Research Center, Oulu University Hospital and University of Oulu, Oulu, Finland; ^14^Research Unit of Medical Imaging, Physics and Technology, Faculty of Medicine, University of Oulu, Oulu, Finland; ^15^Department of Diagnostic Radiology, Oulu University Hospital, Oulu, Finland; ^16^Institute of Rehabilitation, Jyväskylä University of Applied Sciences, Jyväskylä, Finland

**Keywords:** video games, exercise therapy, kinematics, musculoskeletal system, physical therapy, rehabilitation

## Abstract

**Introduction:**

There is limited understanding of how older adults can reach kinematic goals in rehabilitation while performing exergames and conventional exercises, and how similar or different the kinematics during exergaming are when compared with conventional therapeutic exercise with similar movement. The aim of this study was to describe the movement characteristics performed during exercise in custom-designed exergames and conventional therapeutic exercises among patients who have undergone unilateral total knee replacement (TKR). In addition, the secondary aim was to assess the relation of these exercise methods, and to assess participants' perceived exertion and knee pain during exergaming and exercising.

**Materials and methods:**

Patients up to 4 months after the TKR surgery were invited in a single-visit exercise laboratory session. A 2D motion analysis and force plates were employed to evaluate movement characteristics as the volume, range, and intensity of movement performed during custom-designed knee extension-flexion and weight shifting exergames and conventional therapeutic exercises post TKR. The perceived exertion and knee pain were assessed using the Borg Rating of Perceived Exertion and Visual Analog Scale, respectively.

**Results:**

Evaluation of seven patients with TKR [age median (IQR), 65 (10) years] revealed that the volume and intensity of movement were mostly higher during exergames. Individual goniometer-measured knee range of motion were achieved either with exergames and conventional therapeutic exercises, especially in knee extension exercises. The perceived exertion and knee pain were similar after exergames and conventional therapeutic exercises.

**Conclusions:**

During custom-designed exergaming the patients with TKR achieve the movement characteristics appropriate for post-TKR rehabilitation without increasing the stress and pain experienced even though the movement characteristics might be partly different from conventional therapeutic exercises by the volume and intensity of movement. Physical therapists could consider implementing such exergames in rehabilitation practice for patients with TKR once effectiveness have been approved and they are widely available.

## Introduction

Total knee replacement (TKR) is a surgical treatment aiming to reduce pain and to restore knee joint function in severe osteoarthritis common seen among older adults (Bruyère et al., 2019). Therapeutic exercise managed by the physical therapist typically begins on the day of the surgery and is intended to reduce swelling and to increase muscle strength, mobility, balance and movement symmetry and the range of motion (ROM) in the operated knee (Jette et al., 2020). Enhanced therapeutic exercise after discharge from the hospital is essential to improve functional performance (Papalia et al., 2020).

An alternative method to conventional therapeutic exercise for patients with TKR is gamified exercising (Wang et al., 2019), also referred to as active video gaming or exergaming (Oh and Yang, 2010). While exergaming, the technology follows the player's reactions and movements, such as the extension and flexion of the knee, which affect the course and outcomes of the exergame. With this motion tracking technique, exergames can be tailored to a desired exercise movement, such as knee extension movement (Pirovano et al., 2016). Both commercially available and custom-designed exergames have been used in physical rehabilitation in older adults (Skjæret et al., 2016). Studies, which have evaluated the realization of movement during exergaming have shown that similar exergames (Skjæret-Maroni et al., 2016; de Vries et al., 2018) or different levels of difficulty in a single exergame (Skjæret-Maroni et al., 2016) may lead to significantly different movements, and movements trained in the exergames may poorly meet all targeted aspects of the specific rehabilitation goal (Tahmosybayat et al., 2018).

However, there is limited understanding of kinematics during exergaming and how exergaming relates to conventional therapeutic exercises. Because of this lack of knowledge, there are limited possibilities to design specific and progressive exergame-based therapeutic exercises more broadly in rehabilitation (Pirovano et al., 2016). Further, this knowledge will determine whether appropriate exergames can be used in intervention studies evaluating the effectiveness of exergaming. Therefore, the objective of this study was to investigate the movement characteristics that patients with TKR performed during custom-designed exergames developed for postoperative rehabilitation. The following research questions were addressed: (1) What movement characteristics patients with TKR achieve while exergaming? (2) Do movement characteristics of custom-designed exergames relate with movement characteristics of conventional therapeutic exercises in patients with TKR?

## Materials and methods

### Study design

This study employed 2D motion analysis and force plates to evaluate movement characteristics performed during custom-designed knee extension-flexion and weight shifting exergames and conventional therapeutic exercises post TKR. Participants up to 4 months after the TKR surgery (*n* = 7) performed conventional therapeutic exercises and exergames sequentially on a single-visit exercise laboratory session. The outcomes were the volume, range and intensity of movement, and perceived exertion and knee pain during exercising. This pilot study is part of the “Business Ecosystems in Effective Exergaming” project (BEE) and “Gamification in Knee Replacement Rehabilitation” randomized controlled trial (BEE-RCT, ClinicalTrials.gov Identifier: NCT03717727) (Aartolahti et al., 2022). This study was approved by the Ethics Committee of the Central Finland Health Care District (register number 4U/2018) and had hospital research permission from the Central Finland Central Hospital. Prior to participation in the study, eligible patients gave their written informed consent.

### Participants

Patients undergoing TKR were recruited during the hospital pre- or postoperative outpatient visit. The inclusion and exclusion criteria are presented in Table 1.

**Table 1:** Inclusion and exclusion criteria.

**Inclusion criteria**	**Exclusion criteria**
Interested in participating in the study.	Revision or complications related to the TKR surgery.
Live in the region of the city of Jyväskylä.	Chest pain during exercise or other physical exertion.
60–75 years of age.	Unreasonable shortness of breath.
Primary unilateral TKR*	Seizures of unconsciousness, fainting or dizziness.
Mechanical axis of the limb in varus.	Heart medication.
Normal vision with or without eyeglasses.	
No rheumatoid arthritis or other inflammatory joint disease.	
No fracture or other biomechanical disruptions that has affected the lower limb within 1 year before surgery.	
No diagnosed memory disorder.	

^*^Surgical decision made or <3 months after surgery.

### Procedure

Participants attended one 2-hour test session held in the exercise laboratory of the University of Jyväskylä before 4 months had elapsed since their TKR. At the beginning of the test session, participants completed a Western Ontario and McMaster Universities Osteoarthritis index (WOMAC) (McConnell et al., 2001) questionnaire to assess pain (score 0–20), stiffness (score 0–8) and physical function (score 0–68) during the previous 7 days and a visual analog scale (VAS) (Jensen et al., 2003; Thong et al., 2018) to assess knee pain (score 0–100) during the past 24 h. After completing the WOMAC and VAS, the knee joint ROM of the operated knee was measured using a goniometer (Gogia et al., 1987) while the participant was in a supine position. Thereafter, participants performed exercise protocols; first, participants performed conventional post-TKR exercises, and second, custom-designed exergames were performed immediately after those exercises (Table 2).

**Table 2:** Exercise protocols used in the study: (1) conventional therapeutic exercises and (2) custom-designed exergames.

**Exercise**	**Exercise** **position**	**Exercise description**	**Movement during exercise**	**Primary knee movement**
**Conventional therapeutic exercises**				
Ankle pumps*	SU	Bending and straightening of ankles	Flex and extend	NA
Extension supine I*	SU	Pressing the back of the operated knee against the treatment table for 5 s while ankles are flexed	Flex and extend	Ext
Flexion supine*	SU	Bending the operated knee by sliding the foot along the treatment table	Flex and extend	Flx
Extension supine II*	SU	Straightening the operated knee and maintaining the muscle tension for a few seconds while ankle is flexed and back of the operated knee is on half round bolster	Flex and extend	Ext
Extension supine III*	SU	Lifting the straightened operated lower limb up from the treatment table and maintaining the muscle tension for a few seconds while the ankle is flexed	Flex and extend	Ext
Flexion sitting^†^	SI	Bending the operated knee as much as possible (foot may be slide along the floor)	Flex and extend	Flx
Extension sitting^†^	SI	Straightening the operated knee and maintaining the muscle tension for a few seconds while ankle is flexed	Flex and extend	Ext
Extension standing I	ST	Raising heels until standing on toes	Flex and extend	Ext
Extension standing II	ST	Taking a step forward with the operated lower leg, straightening the knee and transferring the weight on the leg	Weight transfer	Ext
Flexion standing^†^	ST	Bending the operated knee and maintaining the muscle tension while keeping thighs next to each other	Flex and extend	Flx
**Custom-designed exergames**				
**Knee extension-flexion exergames**				
Rowing Game^†^	ST	Bending the operated knee to row a boat	Flex and extend	Flx
Cave Game^†^	SI	Straightening and bending the operated knee to catch flies	Flex and extend	Ext
Intruders^†^	SI	Straightening and bending the operated knee to shoot zombies	Flex and extend	Ext
**Squatting exergames**				
Squat Pong^†^	ST	Squatting and raising heels until standing on toes to hit the ball with a tennis racket	Squat	Flx
Pick Up^†^	ST	Squatting to pick up vegetables	Squat	Flx
**Weight shifting exergames**				
Bubble Runner^‡^	ST	Transferring the weight laterally from foot to another to blow up balloons on the road	Weight transfer	Ext
Hat Trick^‡^	ST	Transferring the weight laterally from foot to another to throw objects to a sombrero	Weight transfer	Ext
**Piloting exergames**				
Brick Breaker	SI	Move lower limb laterally to catch falling objects	Hip Abd/Add	NA
Toy Golf	ST	Balance, golf swing, and squatting to give boost to a golf ball	Balance control	Flx
Hiking	ST	Marching to move forward on a trail	Marching	Flx

*SU, Supine in the treatment table; SI, Sitting in the chair; ST, Standing; NA, Not Applicable; Ext, Extension; Flx, Flexion; Abd/Add, Abduction/ Adduction*.

^*^Acted as warm-up exercises.

*^†^Included in motion analysis*.

*^‡^Included in force plate analysis*.

*Conventional exercises and exergames are presented in the order in which participants performed them during a test session*.

#### Conventional therapeutic exercises

The conventional post-TKR exercises (later referred to as conventional exercises) included 10 exercise following instructions of South West Finland Health Care District (South West Finland Health Care District, 2015). Exercises aimed to increase blood circulation, muscle strength and knee ROM of the operated lower limb. Participants were instructed to perform 10 repetitions in each conventional exercise at their own pace. This meant that the duration of each exercise varied both individually and between participants, as the participants could choose to perform repetitions at different paces. For example, knee flexion exercise sitting at a slow pace and standing at a faster pace.

#### Custom-designed exergames

Ten custom-designed exergames (Futuristic Interactive Technologies research group, Turku GameLab, Turku University of Applied Sciences, Finland) used in this study were developed using a Unity game engine (Unity Technologies, USA). The development was made in various test-generate cycles following user-centric design principles and Hevner's IS Research Framework (Hevner et al., 2004). The hardware used was a Kinect 2 motion sensor (Microsoft, USA) and a laptop computer (Micro-Star International, Taiwan) connected to a flat screen television (43 inches). Each exergame included a storyline, which explained the goal of the game, and participants' body movements acted as a game controller. These movements tracked by the Kinect sensor were transferred on the screen to move the avatar. Four exergames (the Cave Game, Rowing Game, Intruders and Squat Pong) included a pre-calibration that scaled the maximum ROM of the participant's operated knee prior to exergaming so that exercising occurred according to the participant's current mobility limitations. Game duration was based on maximum repetitions (the Cave Game), maximum time (the Pick Up, Bubble Runner, Hat Trick, Brick Breaker, Hiking and Toy Golf) or a combination of maximum repetitions and time (the Intruders, Rowing Game and Squat Pong). The combination of repetitions and time means that the game could end after the maximum number of repetitions specified for game, but no later than after the maximum time specified for game. Participants were instructed to perform repetitions according to games' storylines for their duration.

Knee extension-flexion exercises and exergames were performed only with the operated lower limb. Participants were allowed to have breaks between conventional exercises and exergames. Participants took support from the back of the chair when they had to stand on one leg during the performance (the Flexion standing and Rowing Game). More detailed information of conventional exercises and exergames are presented in the protocol of the BEE-RCT (Aartolahti et al., 2022).

### Measurements

#### Movement characteristics

##### Instrumentation

Video data to evaluate knee 2D-kinematics from the sagittal plane during exercising were recorded with a Sony RX-10 III camera (Sony Corporation, Japan) (Norris and Olson, 2011; Schurr et al., 2017). Video recording was continuous throughout the performance of three conventional exercises (the Flexion sitting, Extension sitting and Flexion standing) and exergames (Table 2). Passive markers for 2D motion analysis were placed at participants' joint axes of shoulder (greater tubercle of the humerus), hip (greater trochanter of femur), knee (lateral epicondyle of femur) and ankle (lateral malleolus of fibula). Around these points, the knee and hip joint angles were determined. A camera was placed on a tripod seven meters from the participant so that the camera was facing the participant's lateral side of the body while he or she was standing on force plates. The knee extension-flexion exergames and the conventional exercise were recorded from the operated side and the squatting exergames from the left side. The optical axis of the camera was positioned at the height of the participant's pelvis. At the beginning of each test session, calibration was performed by using a rectangular calibration frame (200.0 × 110.8 cm). The camera's frame rate was 50 frames per second (50 Hz) and the shutter speed was 1/1,250 s.

A force measure to represent how evenly the participants stood still and how much participants leaned on the operated lower limb during exergaming was captured using Vicon Nexus 2 (version 2.7 & 2.8) software (Vicon Motion Systems Ltd, UK), while the participant stood with one foot on each of the two AMTI MiniAMP MSA-6 force plates (Advanced Mechanical Technology Inc., USA). Force plate data were gathered throughout the performance of weight shifting exergames (Bubble Runner and Hat Trick). Participants were instructed to keep their feet throughout the exergames on force plates, which had fixed placement in the exercise laboratory.

##### Data processing

The motion analysis data was filtered with a 15 Hz Butterworth 4th- order-zero-lag low-pass filter (Schreven et al., 2015). Videos were converted to AVI format with Kinovea 0.8.15 software (Kinovea, 2021), and a sagittal 2D model was created in Vicon Motus 10.0.1 motion analysis software (Vicon Motion Systems Ltd, UK) (Norris and Olson, 2011). Piloting exergames (the Brick Breaker, Toy Golf and Hiking) were performed for future development purposes, but kinematics were not assessed in the present study. Two participants are missing one exergame motion analysis data due to wrong performance (Participant 1: Intruders) and software (Participant 2: Pick Up) issues encountered during exergaming (Supplementary material).

Movement characteristics were identified by a custom-made MATLAB script (R2019a, The MathWorks Inc., Natick, Massachusetts, USA). For the analysis, the active time of exercising was defined as duration from which the passive time, i.e., the time the participant got ready to start the conventional exercise or waited for the exergame to start, was removed.

To standardize the analysis, the limits to identifying a single repetition were determined. By these limits, the calculation algorithm detects only full repetitions and not a small movement near the rest position. First, a knee angle limit was determined for each test subject based on participants' individual active knee ROM measured with a goniometer. All movements across this limit were considered active movements. After that, for knee extension and flexion exergames (the Rowing Game, Cave Game and Intruders) and conventional exercises, the limit was defined as half individuals' active knee ROM, and for squatting exergames (the Squat Pong and Pick Up) as a quarter of individuals' active knee ROM. Finally, MATLAB's “findpeaks” function was used to define the repetitions and their knee joint ROM from the active movement. For the “findpeaks” function arguments, it was determined that the time between repetitions was set to 0.7 s in the exergames and 2.2 s in the conventional exercises. The minimum peak prominence, which defines the accepted variation between repetitions, was set to 20 degrees.

**Figure 1:**
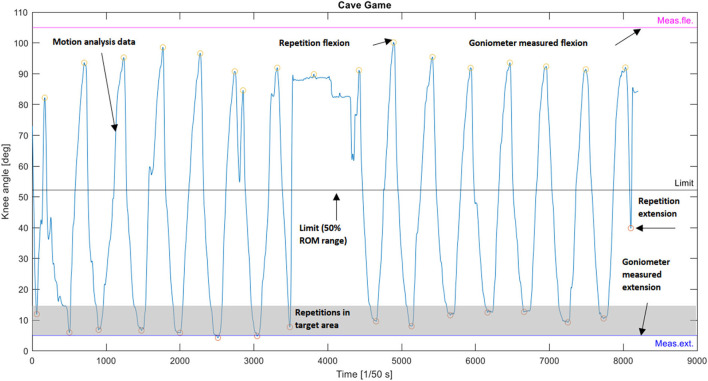
Identification of repetitions from the motion analysis data of the knee extension-flexion exergame (the Cave Game) where knee extension was the primary movement goal.

The *volume of movement* was analyzed by all repetitions completed during the session, repetition rate (repetitions per minute) and duration as the total duration of the gaming or exercising session. In addition, to identify repetitions that reached the active knee ROM (% in active ROM) measured with a goniometer, the angle range, so-called target area, was defined for each test subject. This was set by adding ten degrees to the active knee ROM, which can be considered an error in goniometer measurement (Lenssen et al., 2007) and motion analysis (Elliott et al., 2007). The *range of movement* during the gaming or exercising session was analyzed by knee joint ROM during extension and flexion movement. The *intensity of the movement* was determined using knee angular velocity which was defined as the average angular velocity per second during active movement, and the using knee angle accumulation which was defined as the total angular movement per minute during the entire exercise. An example of repetition identification is presented in Figure 1 and graphs of the analysis are provided in the Supplementary material.

Force plate data was collected at a frequency of 30 Hz and was filtered with a 10 Hz Butterworth 4th- order-zero-lag low-pass filter. The *resultant force* during exergaming on the operated lower limb in relation to the exergame duration was calculated from the two force plate data, which was classified every 10% so that 100% represents the situation where participants were fully leaning on the operated lower limb, and these values are presented as the mean and standard deviation (SD). In addition, the average *resultant force* on the operated and non-operated lower limbs as a percentage was calculated while participants stood still.

#### Knee pain and perceived exertion in exercises

Subjective perceived exertion and knee pain during exercising were assessed using the Borg Rating of Perceived Exertion (Borg, 1982) and VAS, respectively. The assessment took place after performance of the conventional exercises, the knee extension-flexion exergames (the Rowing Game, Gave Game and Intruders), the squatting exergames (the Squat Pong and Pick Up), and the weight shifting (the Bubble Runner and Hat Trick) and piloting exergames (the Brick Breaker, Toy Golf and Hiking).

### Statistical analysis

All statistical analyses were performed in R version 4.0.3 (R Core Team, 2020). The results of the volume and range of movement are reported as the median and interquartile range (IQR), except for repetitions in the target area, which are reported as the mean and SD. The results of the intensity of movement are reported as the mean and SD. The differences in the range and intensity of movement between the conventional exercises and exergames were analyzed using the Wilcoxon signed rank test with the level of significance set at 0.05.

## Results

Eleven individuals were interested in participating in the study. Four patients were excluded because of shortness of breath and dizziness (*n* = 1), change in interest for participation (*n* = 2) and abnormal mechanical axis of the limb (valgus) (*n* = 1). The characteristics of the seven individuals included to participate in the study are presented in Table 3. Participants had a low level of symptoms, good physical function, and mild knee pain during the previous day. None of the participants obtained full knee extension (0°), and only one reached the degree of knee flexion (110°), which is the proposed degree of performing normal daily activities (Rowe et al., 2000).

**Table 3:** Participant characteristics (*n* = 7).

	**Value**
Age (years)	65 (10)
Sex (male/female) (*n*)	1 / 6
Time from TKR (months)	3.5 (2.75)
WOMAC*	
Pain (0–20)	5 (5)
Stiffness (0–8)	2 (1)
Physical function (0–68)^†^	15 (24)
Total (0–96)	21 (28)
Pain in previous 24 h (VAS 0–100)^‡^	42 (45)
**Active ROM of operated knee (goniometer)^**§**^**	
Knee extension	12 (7)
Knee flexion	100 (18)
Resultant force (%) on operated lower limb^|^	48 (2)

*IQR, interquartile range; TKR, Total Knee Replacement; WOMAC, Western Ontario and McMaster Universities Osteoarthritis index; VAS, Visual Analog Scale; ROM, Range of Motion*.

^*^Higher score indicates worse pain, stiffness, and functional limitations.

*^†^For one participant who ticked two options in two questions, values that indicated more severe condition was selected*.

*^‡^Higher score indicates worse pain*.

*^§^Active range of motion in degrees measured in the supine position; Zero degrees (0°) of the knee joint is a position where the knee is fully extended*.

*^|^n = 6*.

*The value is the median (IQR) unless otherwise stated*.

### Movement characteristics

#### Volume

The repetition rate was similar between the exergames (14–15 repetitions/minute) and the conventional exercises (13–15 repetitions/minute), with the exception of the performed repetitions in the Rowing Game (34 repetitions/minute) and the Cave Game (eight repetitions/minute) (Table 4). Variation was observed in the proportions of the repetitions that reached goniometer measured active knee ROM in exergames and conventional exercises. Only in the knee extension performed with conventional exercise (the Extension sitting) each repetition reached measured knee ROM (Table 4).

**Table 4:** Movement characteristics demonstrated by the patients with TKR (*n* = 7) in measured exercises whose movement goal was knee extension and flexion.

	**Volume**				**Range**		**Intensity**	
	**Duration**	**Repetitions**			**Knee joint ROM (**°**)**		**Angular velocity**	**Angle accumulation**
	**(seconds)**	**Total (no)**	**Rate (reps/min)**	**(% in active ROM)**	**Extension**	**Flexion**	**(**°**/s)**	**(**°**/min)**
	**Med (IQR)**	**Med (IQR)**	**Med (IQR)**	**Mean (SD)**	**Med (IQR)**	**Med (IQR)**	**Mean (SD)**	**Mean (SD)**
**Knee extension-flexion exergames (incl. squatting games)**								
Cave game	177 (21)	24 (10)	8 (2)	80 (14)	14 (5)	81 (14)	25.1 (11.0)	1,297.8 (469.4)
Intruders*	70 (85)	19 (7)	15 (8)	96 (3)	11 (5)	95 (20)	74.4 (36.6)	2,769.7 (2,094.5)
Rowing game	62 (8)	31 (14)	34 (11)	83 (37)	19 (9)	93 (11)	67.6 (29.8)	5,021.2 (1,976.8)
Squat pong	118 (32)	28 (8)	14 (3)	0 (0)	11 (6)	56 (14)	20.1 (9.7)	1,249.5 (606.7)
Pick up*	93 (4)	21 (3)	14 (1)	14 (33)	8 (9)	70 (14)	28.1 (8.9)	1,666.6 (560.5)
**Conventional exercises**								
Extension sitting	36 (15)	10 (1)	15 (6)	100 (0)	11 (5)	79 (13)	35.5 (20.4)	1,973.2 (878.7)
Flexion sitting	42 (20)	9 (3)	13 (5)	97 (8)	62 (11)	94 (13)	20.9 (6.6)	1,071.3 (403.2)
Flexion standing	40 (7)	10 (0)	15 (3)	61 (44)	17 (3)	89 (14)	36.9 (9.6)	2,083.0 (441.7)

*Med, median; IQR, interquartile range; SD, standard deviation; Repetitions; Rate (reps/min), time-proportional rate of repetitions; ROM, range of motion; Repetitions; (% in active ROM), repetitions that reached the ROM measured with a goniometer (presented from the primary knee movement of the exergame/conventional exercise)*.

*°Degree*.

*n = 6.

*Exercises are presented in the order of the exercise protocols (exergames and conventional exercise) and the primary knee movement in the exercise (extension and flexion)*.

#### Range

Participants extended the knee similarly in the Intruders and conventional Extension sitting exercise (both 11°, *p* > 0.05), but significantly less in the Cave Game than in the conventional Extension sitting exercise (14° and 11°, respectively, *p* = 0.018) (Tables 4, 5). Significantly higher knee flexion ROM in the Rowing Game was seen when it was compared to the conventional Flexion standing exercise (93° and 89°, respectively, *p* = 0.018) (Tables 4, 5). The knee flexion ROM in the conventional exercises (89° and 94°) was significantly larger than that in the Squatting exergames, i.e., the Squat Pong (56°, *p* = 0.018) and Pick Up (70°, *p* < 0.05) games (Tables 4, 5).

**Table 5:** Significance levels (*p*-values) for differences in the range and intensity of movement in the exergames and conventional exercises according to the primary movement goal: (A) knee extension and (B) knee flexion.

**(A)**		**Cave Game**		**Intruders**	
	***N***	**ROM**	**Velocity**	**ROM**	**Velocity**
Extension sitting	7	0.018*	0.176	0.345	0.046*
**(B)**		**Rowing Game**		**Squat Pong**		**Pick Up**	
	***N***	**ROM**	**Velocity**	**ROM**	**Velocity**	**ROM**	**Velocity**
Flexion sitting	7	0.237	0.018*	0.018*	0.735	0.028*	0.173
Flexion standing	7	0.018*	0.063	0.018*	0.018*	0.046*	0.046*

*ROM, Knee joint range of motion; Velocity, Angular velocity*.

^*^Wilcoxon signed rank test p < 0.05.

#### Intensity: Angular velocity

In the knee extension exergames, the Intruders game was significantly more intense than the conventional Extension sitting exercise (74.4 and 35.5°/s, respectively, *p* = 0.046) (Tables 4, 5). Significantly higher intensity in the Rowing Game was apparent when compared to the conventional Flexion sitting exercise (67.6 and 20.9°/s, respectively, *p* = 0.018) (Tables 4, 5). Movement in the conventional Flexion standing exercise (36.9°/s) was significantly more intense (*p* < 0.05) than in the Squatting exergames (the Squat Pong and Pick Up) (20.1 and 28.1°/s, respectively) (Tables 4, 5).

#### Intensity: Resultant force

**Figure 2:**
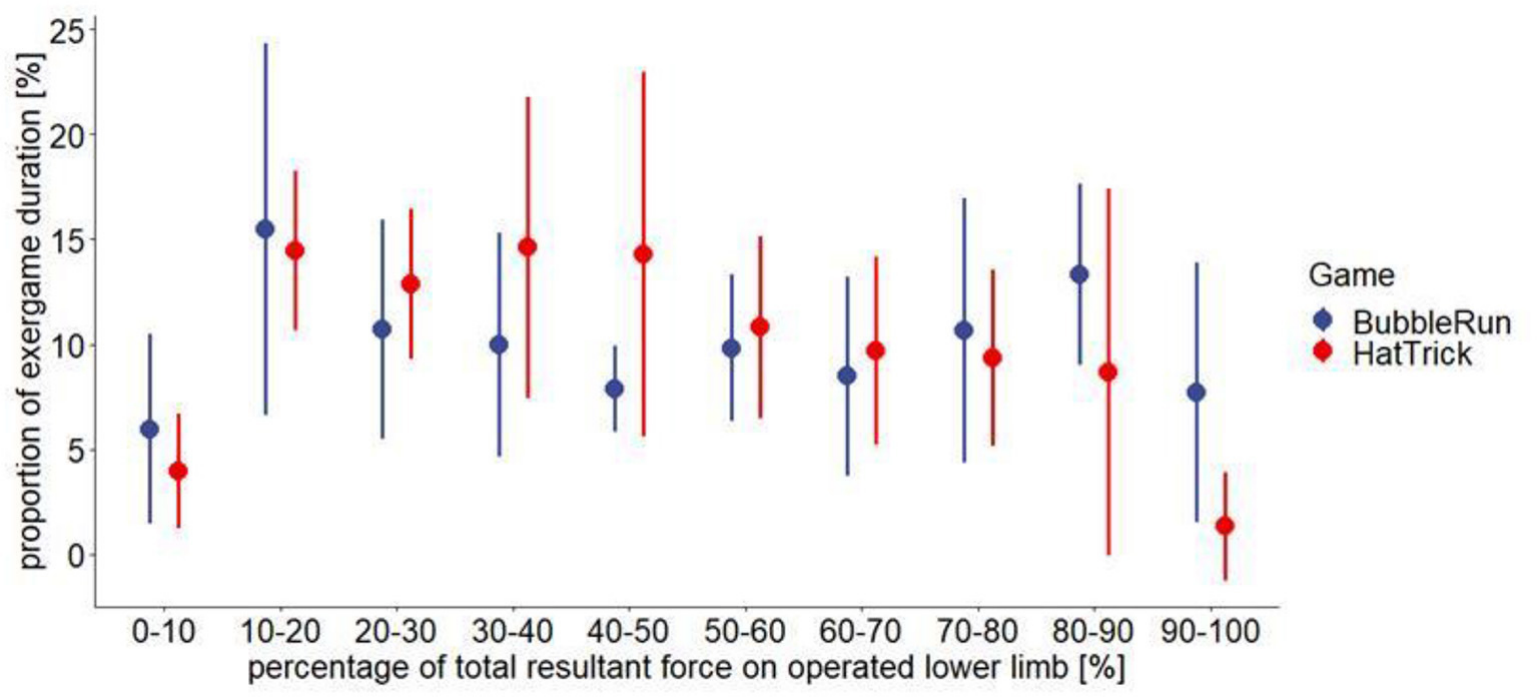
The resultant force (the mean and SD) produced by the operated lower limb (*n* = 7) during weight shifting exergames (the Bubble Runner and Hat Trick).

Visual inspection of movement during the weight shifting exergames (the Bubble Runner and Hat Trick) suggests that the majority of the resultant force was produced by the non-operated lower limb (Figure 2).

### Knee pain and perceived exertion in exercises

Participants experienced mild knee pain after performing conventional exercise (median: 8; IQR: 6), knee extension-flexion exergames (15; 12), squatting exergames (7; 17), and weight shifting and piloting exergames (4; 4). The perceived exertion was “very light” after the weight shifting and piloting exergames (10; 4), “light” after the conventional exercises (12; 5) and the knee extension-flexion exergames (11; 3), and “somewhat hard” after the squatting exergames (13; 4).

## Discussion

The study demonstrated some of the expected results regarding individual and intergame variation during exergaming, which has also been observed in other studies (Skjæret-Maroni et al., 2016; de Vries et al., 2018). The present study observed a higher number of repetitions performed in the exergames, which was due to longer exercise duration. When repetitions were proportional to duration of exercise, repetition rate was more equivalent between exergames and conventional exercises. Moreover, when exergames were designed to follow more closely the movement of the conventional exercise, the measured movement characteristics were indeed mainly similar. Smaller knee ROM during squatting exergames, in turn, may be a result of the simultaneous function of multiple joints and muscle groups of the lower body required during squatting and thus is a more holistic exercise than isolated knee extension or flexion exercise. Nevertheless, the movement characteristics of exergames with squatting actions are worth studying and can be considered a good addition to the postoperative rehabilitation of patients with TKR (Zeni et al., 2013; Tsuzuku et al., 2018), which is also supported by the results of low perceived exertion and knee pain during the squatting exergames.

When clinically evaluating the study results of the knee ROM during the knee extension-flexion exergames, 80–96% of repetitions performed during exergaming reached the target area calculated from the goniometer-measured knee ROM. Participants even extended the knee over the goniometer-measured extension in the Intruders game but did not achieve goniometer-measured flexion in the Rowing Game. However, repetitions in the target area and knee flexion degree were larger in the Rowing Game than in the conventional knee flexion exercise with the same movement. These outcomes are positive indicators of successful implementation of conventional exercise as a game (Pirovano et al., 2016), although it is unknown whether the observed significance levels in ROM during exercise actually translates to meaningful clinical difference, and moreover, the repetitions made during exergames and conventional exercises may not be realized fully similarly. For example, due to exergame's storyline, there may be a longer time between repetitions, while it may be fairly constant in conventional exercise. This was seen especially in the motion analysis data of Intruders and Extension sitting exercise.

When evaluating the movement characteristics demonstrated during exergaming, some personal and gaming-related issues that may have an impact on the achievement of exercising goals and adherence should be considered. The player's intentional or unconscious choices during exergaming may negatively affect the number or quality of movements made. This finding has been observed as a learning effect among healthy older adults (Skjæret-Maroni et al., 2016; de Vries et al., 2018), but in patients with TKR, it may also be related to persistent postoperative pain (Beswick et al., 2012) or asymmetry in placing weight on the operated lower limb (Christiansen et al., 2013). For example, despite the exergame's story outline, patients with TKR could choose to predominately lean on to their non-operated lower limb for a longer time, not to perform the repetition, or not to squat to the desired knee flexion angle. Hence, further study into user experiences may provide a benefit for developing more immersive exergames (Dulau et al., 2019).

However, it should be noted that participants included in the current study had low pain during exergaming, and clinical asymmetry between operated and non-operated lower limbs was low while standing still. Consequently, the exergames examined in the present study appear to be suitable and safe to perform for the studied population, and some individuals may even find gaming more fun and a motivating form of exercise (Pyae et al., 2017). It is also worth noting that the incidence of TKRs among younger age groups is increasing, and these patients may be potentially more interested in using exergames in rehabilitation (National Institute for Health Welfare, 2021). In addition, the exergames used in the study were designed so that participants performed both knee extension and flexion movements in one exercise and thus replaced two conventional exercises. These aspects could increase adherence to exercising when the goals of the two exercises may be achieved in one exercise, leading to a shorter duration of exercising sessions reportedly preferred by older adults (Zadro et al., 2019).

To the best of our knowledge, this was the first study specifically to investigate kinematics, perceived exertion, and knee pain during custom-designed exergames and their relation to conventional postoperative exercises after TKR. This type of research is necessary when designing exergames or future randomized, controlled trials investigating the effectiveness of exergames for rehabilitation with specific exercise goals. This knowledge may also lead to the development of better and more customized exergames, the use of which in effectiveness studies is still low (Corregidor-Sánchez et al., 2021). Moreover, in the future, when exergames are developed further, physiological measurements, such as muscle activation, heart rate and blood pressure, could be studied to gain an even better understanding of physical loading during exergaming (da Cruz et al., 2020; Willaert et al., 2020). Finally, game developers may consider how exercise instructions may be added or merged to exergames (Pirovano et al., 2016) and provide, for example, the possibility for study participants or persons in the rehabilitation process to use exergames without supervision.

The strengths of this study were valid and appropriate instrumentation, such as custom-designed exergames developed for patients with TKR and 2D motion analysis (Norris and Olson, 2011; Schurr et al., 2017), and the custom-made movement characteristic identification in the data processing. Despite the strengths, two limitations should be considered. First, the test sessions were non-randomized. When participants performed the conventional exercises first and then the exergames, the conventional exercises could even act as a warm-up to exergaming or conversely cause fatigue or pain toward the end, despite the low perceived exertion and pain observed during exergames. To prevent these possible effects, future studies could consider randomizing the order of the exercise protocols. Second, the number of participants was low, but nevertheless, the study showed important results in performing movement characteristics. Future studies with a higher number of participants and therefore a higher number of performed exercise protocols could provide the possibility to evaluate relationships between performed movement characteristics and elapsed time since the TKR. These aspects could be considered to provide additional knowledge to assess the appropriateness of different exergames at various stages of the rehabilitation process as well as progression demands in the exergames.

## Conclusions

The results of this study showed that during custom-designed exergaming the patients with TKR achieve the movement characteristics appropriate for post-TKR rehabilitation without increasing the stress and pain experienced even though the movement characteristics might be partly different from conventional therapeutic exercises by the volume and intensity of movement. Physical therapists could consider implementing such exergames in rehabilitation practice for patients with TKR once effectiveness have been approved and they are widely available.

## Data availability statement

The original contributions presented in the study are included in the article/Supplementary material, further inquiries can be directed to the corresponding author/s.

## Ethics statement

The studies involving human participants were reviewed and approved by the Ethics Committee of the Central Finland Health Care District. The patients/participants provided their written informed consent to participate in this study.

## Author contributions

MJ, AL, SW, TP, NK, SC, JP, KP, ML, RK, TJ, AH, and EA substantially contributed to the conception and design of the work. AL, NK, ML, and EA participated in the development of exergames. MJ, JP, KP, and EA participated in the recruitment of participants. MJ, AL, TP, and EA collected the data. MJ, AL, SW, TP, and EA analyzed and interpreted the data. MJ, AL, and TP drafted the manuscript. All authors commented on the drafted manuscript and critically revised it. All authors read and approved the final manuscript.

## Funding

Financial support as part of BEE research project from Business Finland and Finnish partner companies: SE Innovations Oy (Senior Some Oy), Suunto Oy, Physiotools Oy, GoodLife Technology Oy, Lingsoft Oy, eSeteli Palveluverkko Oy, PN Turku Oy, Ade Animations Design & Effects Oy, Adesante Oy, 4FeetUnder, Intechso and Realmax Oy (grant numbers: 5794/31/2016, 5941/31/2016, 6057/31/2016), and Shanghai Jiao Tong University (YG2017MS62).

## Conflict of interest

The authors declare that the research was conducted in the absence of any commercial or financial relationships that could be construed as a potential conflict of interest.

## Publisher's note

All claims expressed in this article are solely those of the authors and do not necessarily represent those of their affiliated organizations, or those of the publisher, the editors and the reviewers. Any product that may be evaluated in this article, or claim that may be made by its manufacturer, is not guaranteed or endorsed by the publisher.
