# Supplementary material for: Movement characteristics during customized exergames after total knee replacement in older adults
Source: Front Sports Act Living. 2022 Jul 27;4:915210. doi: 10.3389/fspor.2022.915210 (PMC9363837; doi:10.3389/fspor.2022.915210)
Supplement: Supplementary file 1 [file Data_Sheet_1.PDF]

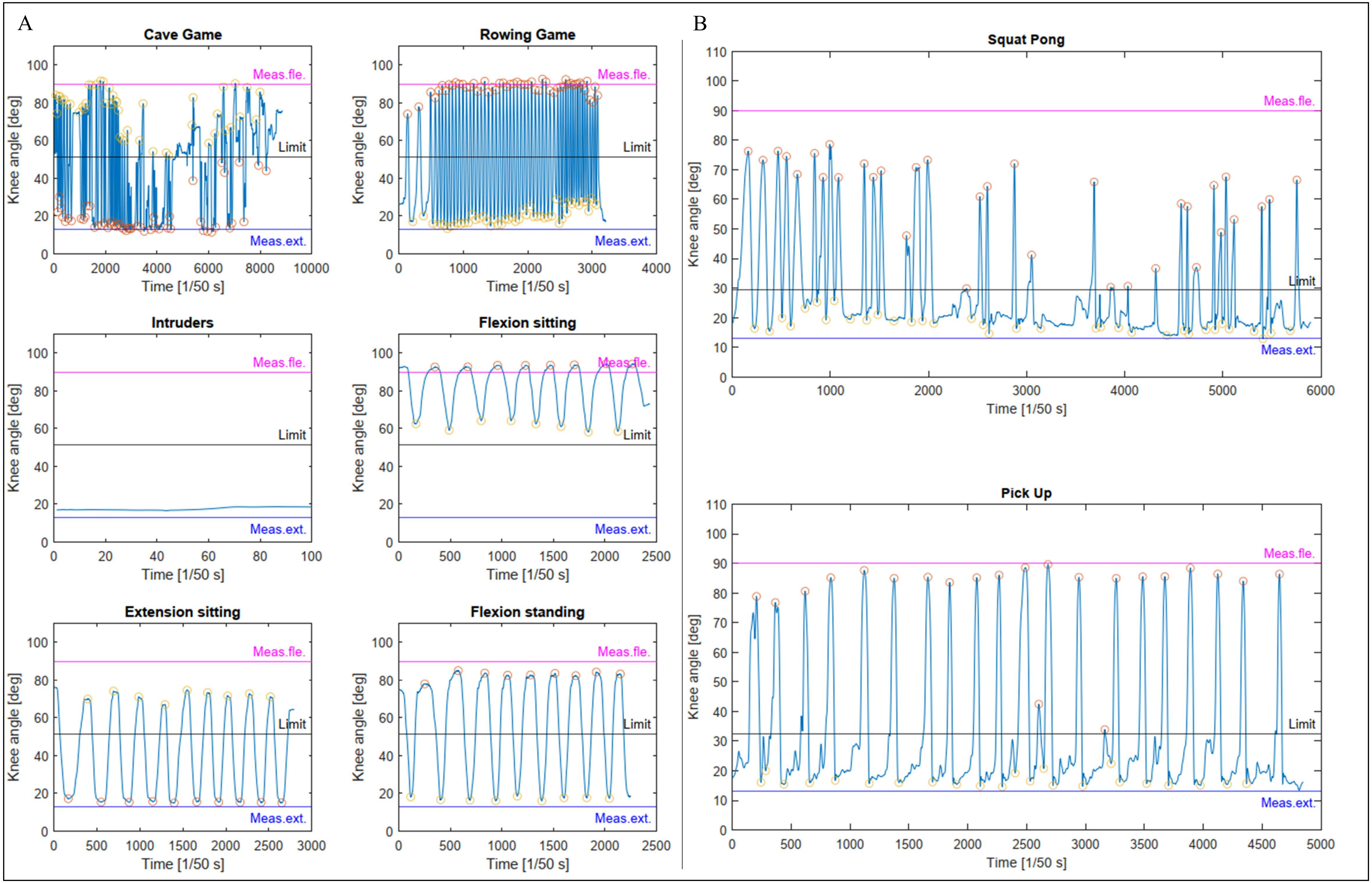

Supplementary Figure 1. Participant 1: Knee angle during knee extension and flexion games (A) and exergames with squatting movement (B).

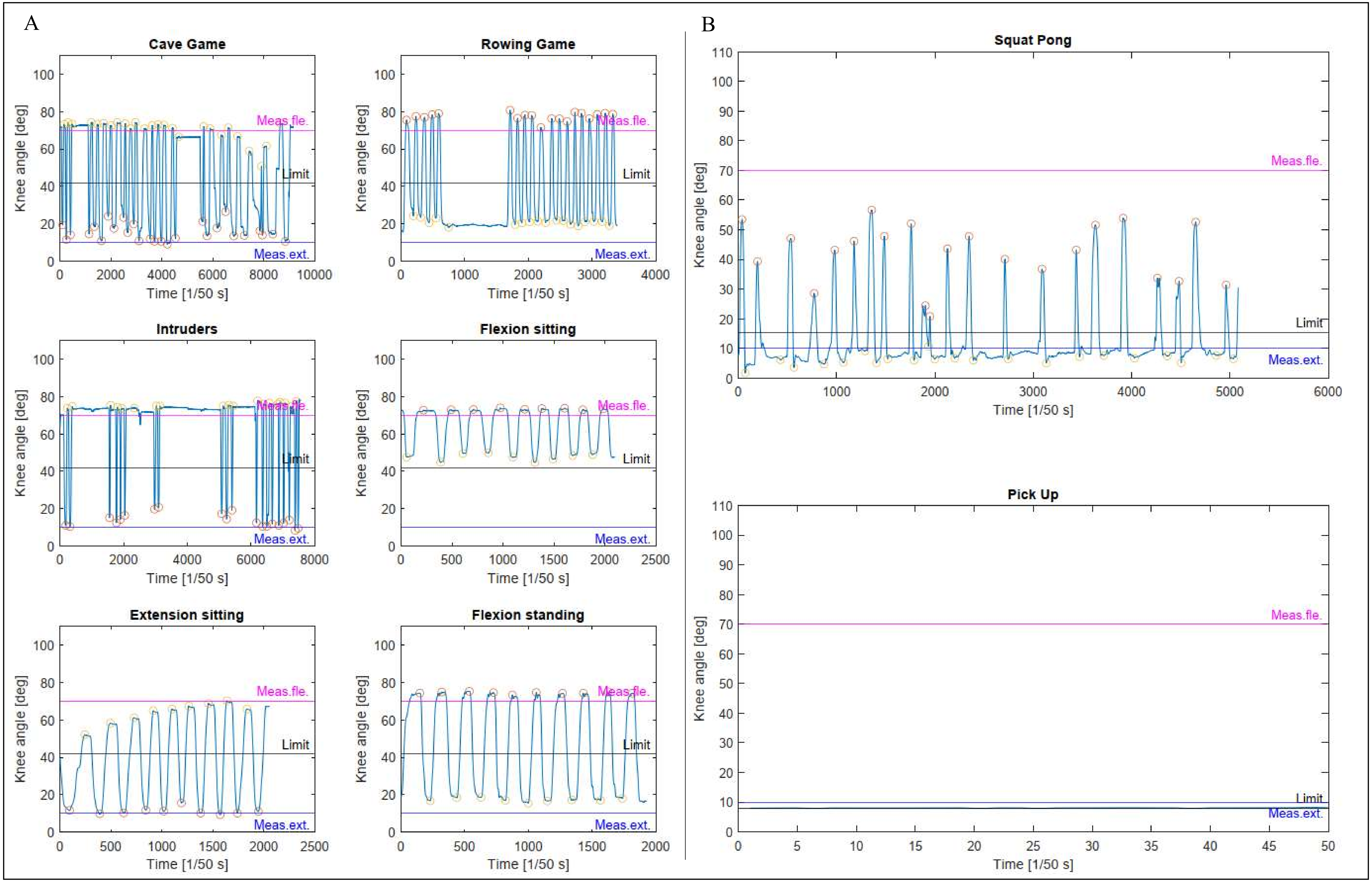

Supplementary Figure 2. Participant 2: Knee angle during knee extension and flexion games (A) and exergames with squatting movement (B).

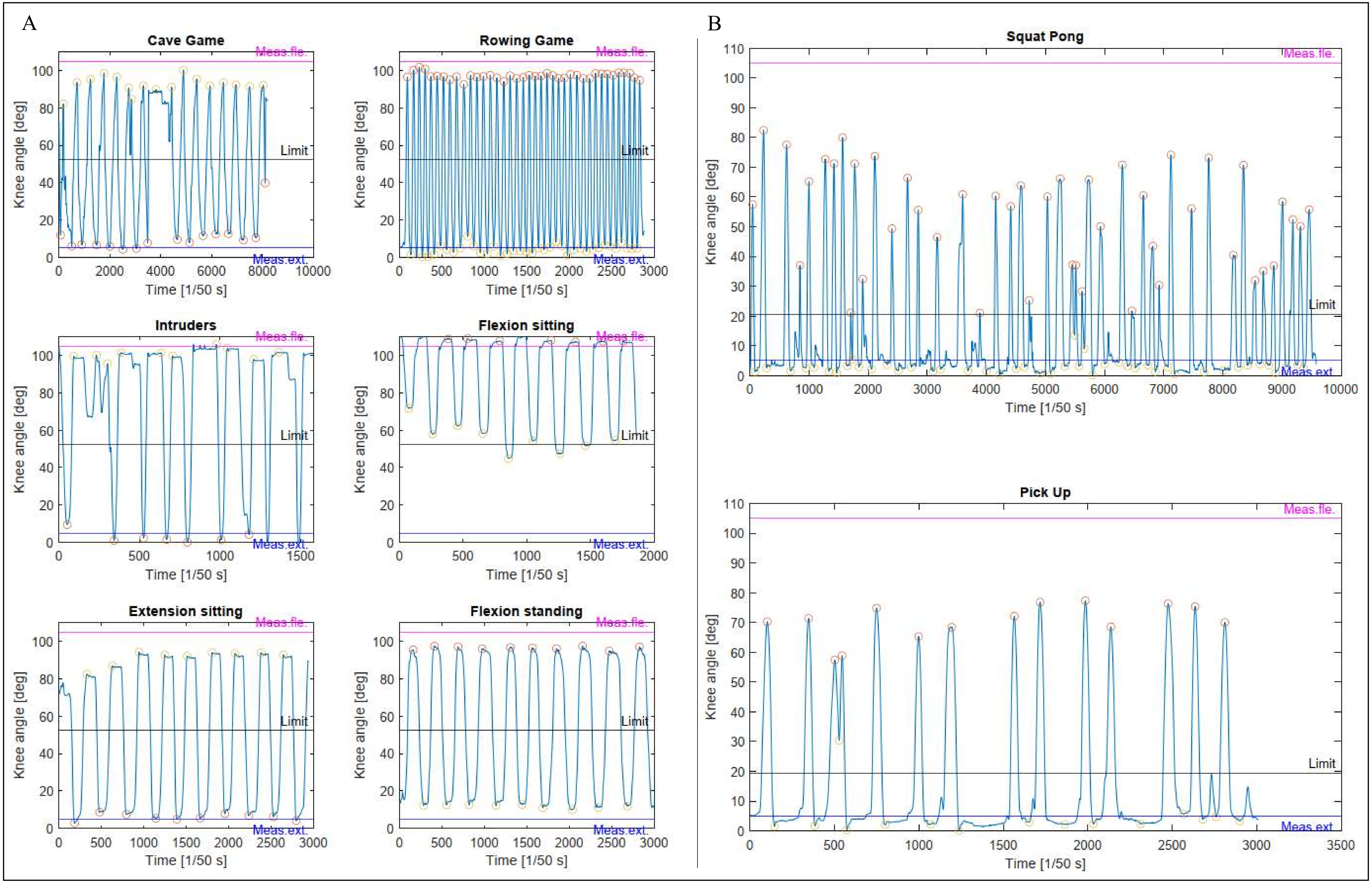

Supplementary Figure 3. Participant 3: Knee angle during knee extension and flexion games (A) and exergames with squatting movement (B).

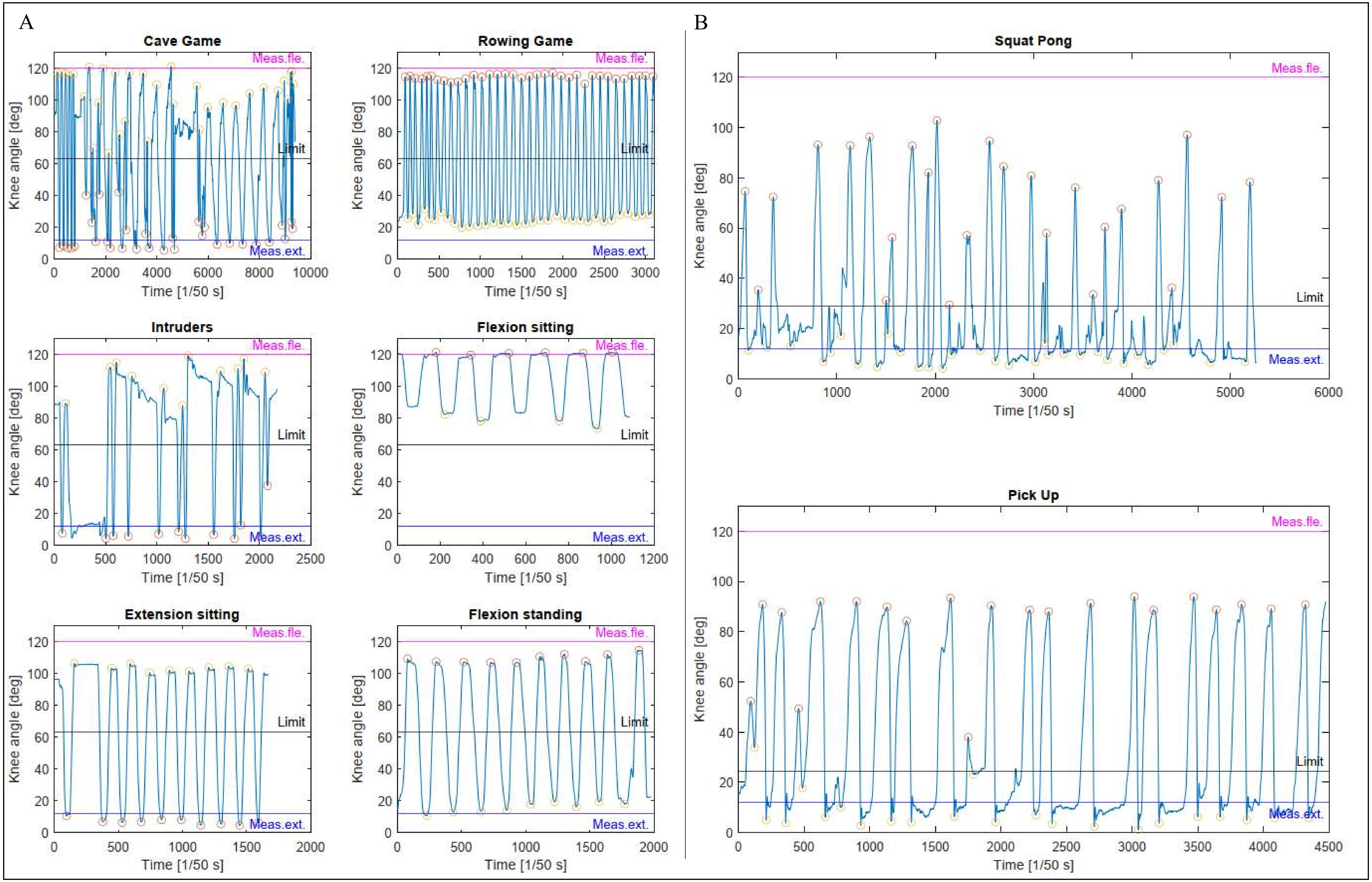

Supplementary Figure 4. Participant 4: Knee angle during knee extension and flexion games (A) and exergames with squatting movement (B).

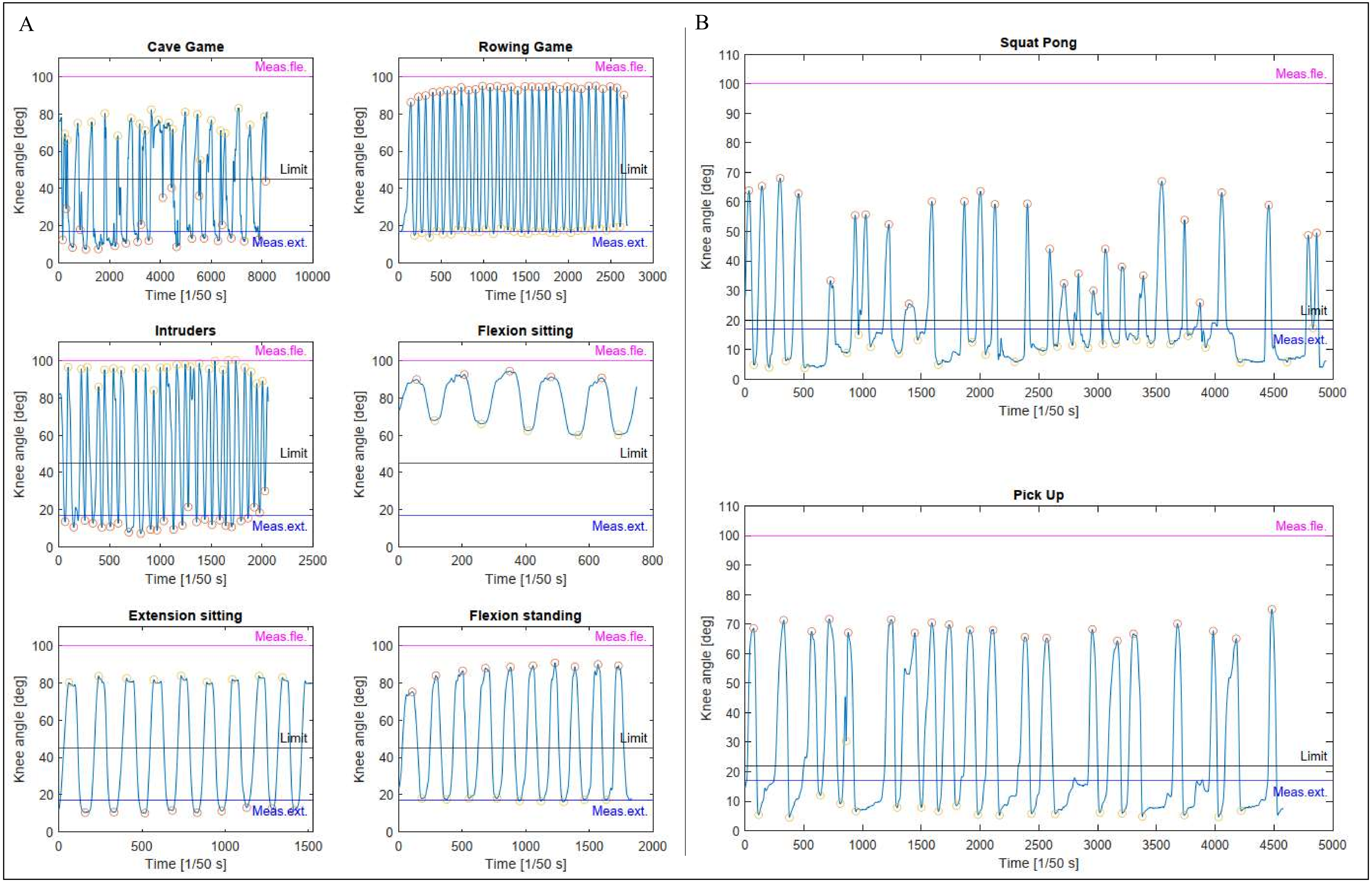

Supplementary Figure 5. Participant 5: Knee angle during knee extension and flexion games (A) and exergames with squatting movement (B).

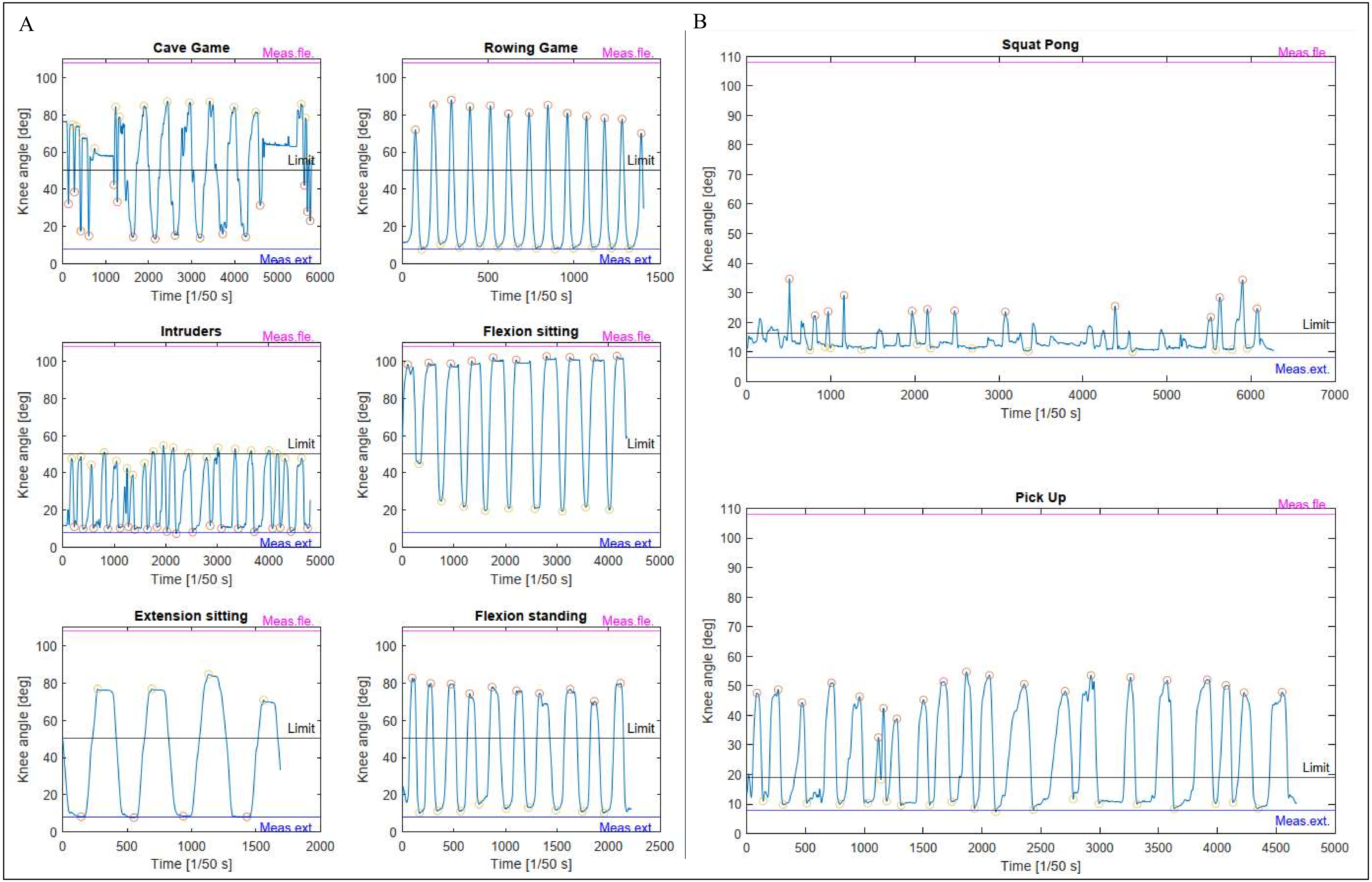

Supplementary Figure 6. Participant 6: Knee angle during knee extension and flexion games (A) and exergames with squatting movement (B).

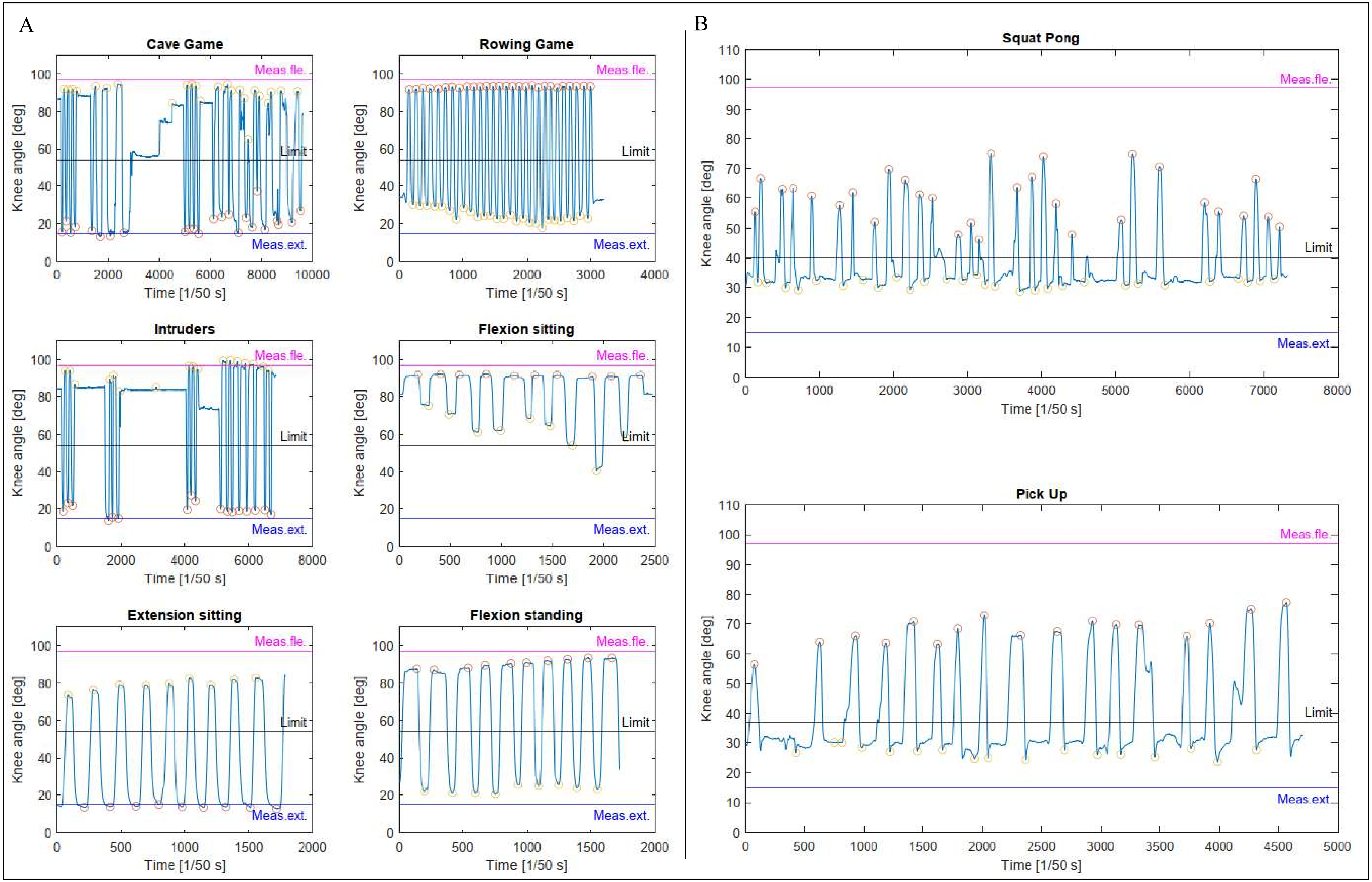

Supplementary Figure 7. Participant 7: Knee angle during knee extension and flexion games (A) and exergames with squatting movement (B).
